# Gender differences in the perception of quality of life during internal medicine training: a qualitative and quantitative analysis

**DOI:** 10.1186/s12909-018-1378-9

**Published:** 2018-11-26

**Authors:** Renata Kobayasi, Patricia Zen Tempski, Fernanda Magalhâes Arantes-Costa, Mílton Arruda Martins

**Affiliations:** 1 Faculty Development Center (CEDEM), School of Medicine of University of Sao Paulo, Sao Paulo, Brazil; 2 Laboratory of Experimental Therapeutics (LIM 20), School of Medicine, University of Sao Paulo, Sao Paulo, Brazil

**Keywords:** Medical residency, Gender bias, Quality of life, Resilience, Empathy, Daytime sleepiness

## Abstract

**Background:**

The higher level of participation by women in medicine may impact this profession’s evolution due to gender differences perceived during medical school, after graduation and during residency. Gender differences regarding quality of life are associated with higher states of anxiety and depression among female physicians.

We aimed to assess gender differences in the perception of quality of life with quantitative methods and to understand further, from the female residents´ point of view, the reasons that may influence the perception of quality of life using qualitative method. Resilience, empathy and daytime sleepiness were also scored.

**Methods:**

We performed a cross-sectional study with first-year internal medicine residents to evaluate self-reported quality of life factors specific to medical residents (VERAS-Q), including empathy (Jefferson Scale of Empathy), resilience (Wagnild and Young Brief Resilience Scale) and daytime sleepiness (Epworth Scale). We explored, from the female residents´ view which factors may influence the perception of quality of life using a focus group method.

**Results:**

In our study, one hundred and nine residents completed the survey: 31 (28.4%) were female and 78 (71.6%) were male. Female residents exhibited significantly lower scores than those of male residents for quality of life in the domains of time management (30.3, females vs 41.1, males; *p* < 0.001), psychology (48.1, females vs 56.7, males; *p* < 0.01) and physical health (42.8, females vs 53.6, males; *p* < 0.05). Female residents also scored higher for daytime sleepiness (13.0, females vs 9.0, males; *p* < 0.001), with pathological scores for daytime sleepiness. No significant gender differences were found in the resilience or empathy scores. The focus group assessment revealed difficulty in concentration and knowledge acquisition, insecurity, feelings of loss, greater critical perception, self-doubt and difficulty in creating effective bonds to support the training period as the main factors involved in the lower perception of quality of life among the women.

**Conclusions:**

In conclusion, female residents had lower scores for quality of life and higher scores for daytime sleepiness. Measures to improve quality of life among female residents during this critical period of medical training might include investing in mentoring to help them better manage their time and encouraging activities that facilitate relationship development.

## Background

Traditionally, medicine has been a male-dominated profession. Despite the increased level of participation by women in the modern medical workforce, in-house training programs continue to mirror the historical model from when few women were in this profession. With the increased female population in medicine, several studies have addressed gender bias and differences regarding perceived quality of life and their important repercussions on mental health, such as states of anxiety and depression among medical students and residents [1–4]. The prevalence of reactive stress symptoms is higher among female residents than among the general population [5].

Some studies analyzing the psychological evolution of stress during medical residency have reported that first-year residents have higher stress levels and more symptoms of depression than second- and third-year residents, who report increased job satisfaction [6–9]. Notwithstanding these findings, little is known about gender-influenced experiences during internal medicine training.

The perception of quality of life has been related to environmental and personal factors. Prior research has shown that resilient medical students exposed to a given level of stress have a better perception of quality of life and of the educational environment [10]. Resilience refers to the ability of individuals to rebound quickly from stressful events and to rebalance themselves when exposed to adverse circumstances, seeing these experiences as possibilities for development and not just as negative situations [11]. In medical residency, numerous stressors such as excessive workload, coping with patient pain, high competitiveness, and pressure for good academic performance contribute to diminish empathetic behavior, impacting the ability to create affective bonds [12, 13]. In several studies, positive relationships are associated with resident well-being [12, 13]. Because of these associations, we evaluated resilience levels and empathy scores in a group of first-year internal medicine residents.

Daytime sleepiness has also been associated with low quality of life [14] In medical residency the prolonged working hours and nigh shifts results in sleep deprivation. Some studies reported that sleep loss affected task performance, learning ability, professionalism, quality of life and personal relationships in medical residents [15–17]. We analyzed daytime sleepiness as a factor that might affect perceived quality of life.

Understanding the factors that might affect the quality of life of female medical residents is fundamental for evaluating the impacts of higher levels of participation by women in medicine. This increased participation may constitute a foundational element of the profession’s evolution, with consequences for medical practice.

Most previous studies have examined medical resident quality of life using general instruments [18]. To our knowledge, no study has compared quality of life between genders using an instrument specific to the medical residency environment. In this study, we used a questionnaire specifically designed to evaluate quality of life in medical residents (VERAS-Q) [19].

This study aims to evaluate gender differences in the perception of medical resident quality of life with quantitative methods in a cohort of first-year medical residents and to understand which factors influence female residents´ perception of quality of life during internal medicine training with qualitative methods. Additionally, resilience, empathy and sleepiness scores were evaluated.

## Methods

The study was designed as a mixed-method approach. The study combined a quantitative analysis based on self-reported questionnaires for the evaluation of quality of life specific to medical residency (VERAS-Q), daytime sleepiness (Epworth Scale), resilience (Wagnild & Young Brief Resilience Scale) and empathy (Jefferson Scale of Empathy), as well as a qualitative analysis with focus group assessment.

### Local structure of the internal medicine residency program and participants

After admission to the internal medicine residency program of the School of Medicine of the University of São Paulo, residents divide themselves into 12 groups of 5 residents. The rotations extend over different periods; two are 2 months long (outpatient and wards at the University São Paulo Hospital) and the others are 1 month long (emergency medicine, emergency cardiology, and the critical care and coronary units). Two periods lasting 1 month each are scheduled electives and the resident has the opportunity to choose a clinical subspecialty or to pursue an external elective. The vacation period lasts 1 month.

Of all the first-year internal medicine residents (*n* = 120) from 2015 and 2016, 110 participated in the Objective Structured Clinical Examination (OSCE) activity, and 109 (90.83%) completed all the questionnaires selected for the study.

### Instruments

#### Socio-demographic assessment

A questionnaire was developed to collect data on age, gender, with whom he/she resides, the number of hours worked outside the residency program, intended specialty and time since returning from vacation.

#### Quality of life assessment

The VERAS-Q specific for residents is a questionnaire created to evaluate the quality of life of residents in health professions [19, 20]. It contains 51 statements on a 5-point Likert scale divided into four domains (time management, psychological, physical health and educational environment) and a global score. There is no cut-off, and the score increases with higher quality of life.

For the time management domain, we assessed the time management ability of residents and whether they were able to enjoy or dedicate time to activities beyond those of the residency program. For the psychological domain, we evaluated positive and negative feelings, concentration, support, self-esteem and demands. For the physical health domain, we evaluated attention to personal health, quality of sleep, leisure, physical activities and satisfaction with appearance. For the educational environment domain, we evaluated the residency program organization, and relationships with colleagues, teachers and the institution [19]. Examples of the items include “my medical residency deprives me of some personal appointments” and “I haven’t been able to properly concentrate lately”.

In the present study, the Cronbach’s alpha value for this scale was 0.90.

#### Sleepiness assessment

The Sleepiness Scale Rating of Daytime Sleepiness developed by Epworth and validated by Murray Johns is a self-administered instrument. We used the version translated and validated for Brazilian Portuguese [21–24]. This assessment contains eight statements, responses are scored as 0 to 3 and total scores range from 0 to 24; it is designed to measure the tendency for sleepiness in everyday situations, for example, “as a passenger in a car for an hour without a break” or “lying down to rest in the afternoon when circumstances permit”. Scores higher than 10 indicate excessive daytime sleepiness and represent pathological scores; scores above 16 are considered very pathological [21–23]. In the present study, the Cronbach’s alpha value for this scale was 0.82.

#### Resilience assessment

The Brief Resilience Scale of Wagnild and Young (RS-14) [25–27] contains 14 items and includes the following areas: self-reliance, meaning, equanimity, perseverance and existential aloneness. Examples of items include “I am determined” and “when I make plans I follow through with them”. The RS-14 answers are scored on a Likert scale from 1 to 7 (1 = strongly disagree and 7 = strongly agree) with a possible total score of 14 to 98. The higher the score, the higher the resilience level. We used the version translated and validated for Brazilian Portuguese [28]. In the present study, the Cronbach’s alpha value for this scale was 0.86.

#### Empathy rating

A Brazilian Portuguese validated version of the Jefferson Scale of Physician Empathy (JSE) questionnaire was used to assess the empathic behavior of medical residents [29, 30]. The JSE consists of 20 items scored on a seven-point Likert scale with the scale score calculated based on the sum of the item scores. Examples of the items include “I believe that empathy is an important therapeutic factor in medical treatment” and “it is difficult for a physician to view things from the patient’s perspective”. In the present study, the Cronbach’s alpha value for this scale was 0.77.

#### Procedures

The quantitative analysis was performed before the beginning of the Objective Structured Clinical Examination (OSCE) [31–33], which is administered every year to first-year internal medicine residents during the eleventh month of their rotation.

Immediately before the beginning of the evaluation, the residents were given questionnaires interrogating socio-demographic factors, quality of life specific to medical residency (VERAS-Q), daytime sleepiness (Epworth scale), resilience assessment using the Wagnild & Young Brief Resilience Scale (RS-14) and empathy behavior analysis using the Jefferson Scale of Empathy (JSE).

The questionnaires were identified by the residents’ registration number to reduce the effect of fear of identification of the responses and to minimize a socially accepted response pattern to the questionnaires.

This study received approval from the Research Ethics Committee of the University of São Paulo. The residents received information about the research objectives and signed a request for free and informed consent before the administration of the OSCE.

#### Statistical analysis

Descriptive statistics were used to determine the distribution of the residents’ response scores for the various questionnaires (mean, median, standard deviation, 25th and 75th percentiles, interquartile range (IQR) and the distribution of the scores in the domains of each questionnaire (frequencies).

The Mann-Whitney U-test was used to compare measures of the central tendency of the domains analyzed according to gender. To analyze individual VERAS-Q items, we calculated the frequency of participants who responded positively to the situation described in each item. The Fisher test was used to compare each statement by gender, and we presented items with the ratio and absolute gender differences for positive perspectives.

On the RS-14 questionnaire, the analysis of the distribution of responses per statement was evaluated as follows: the responses of totally agree and agree were categorized as “Agree” and the other alternatives, indifferent, totally disagree and disagree, were considered “Disagree + Indifferent”, thereby polarizing positive versus negative alternatives. The Fisher test was used to compare each statement by gender.

We established the level of statistical significance as 0.05. All statistical analyses were performed using SPSS Statistics for Windows, Version 22.0 (released 2013, IBM Corp, Armonk, NY).

#### Focus group and qualitative analysis

The focus group analysis of female residents was performed to generate an in-depth understanding of the differences found in quantitative analysis and to raise proposals to improve quality of life for future internal medicine residents. The methods of the focus group were attendant to the Critical Appraisal Skills Program (CASP) [34]. All female residents that completed all the questionnaires were invited to participate by email. Participation was voluntary, with free and informed consent. Ten female residents accepted the invitation. The sessions were led by a coordinator and an assistant and lasted 90–120 min. All discussions were recorded in audio and were associated with the observer’s notes. The main topics discussed were *“Perceptions of quality of life for female medical residents”, “Factors that increase residents’ quality of life”, “Factors that decrease medical resident quality of life”, “Factors that differentially affect the medical residency quality of life for men* versus *women*”, and *“Proposals to improve quality of life for future internal medicine residents”.* The qualitative data analysis followed traditional content analysis methods, that is, using transcripts of the recorded interviews, free reading, highlighting subjects by relevance and repetition, discussion with the research group, and a descriptive presentation of the results using quotes from the residents’ narratives [34–38]. The analysis of the results started with a free reading of the transcribed text by two independent researchers. The first reading aimed at impregnating researchers with the study topic without any intention of categorization. In the second reading, the researchers categorized themes and derived issues independently. The researchers’ results were combined by similarity of meaning, and a third researcher reviewed the analysis. The results were divided into analytical categories, items and examples of narratives.

## Results

Of the 120 first-year residents in internal medicine, 110 participated in the OSCE and 109 residents completed the survey for a response rate of 90.83% of all the internal medicine residents. The participating residents comprised 31 (28.4%) females and 78 (71.6%) males with a median age of 26.00 years (IQR = 2) and an age range of 23 to 33 years.

According to the socio-demographic analysis, the number of weekly hours worked outside the residency program was significantly higher for male residents than female residents (median 12.0, IQR 6.0–12.0, males vs median 6.0, IQR 0.0–12.0, females; *p* < 0.05).

Female residents had significantly higher scores for daytime sleepiness than male residents (13.0, females vs 9.0, males; *p* < 0.001). The values obtained for females are classified as pathological somnolence. Regarding the percentage difference, 74.2% of female residents were classified as pathologically somnolent versus 37.2% of male residents (*p* < 0.01). No significant gender differences were observed for the resilience or empathy scores (Table 1).

**Table 1:** Resilience, sleepiness and empathy scores of medical residents according to gender

Questionnaire	Gender		*P*
	Male (*n* = 78)	Female (*n* = 31)	
Resilience			
Median	81.00	77.00	0.114
IQR	14	13	
P25-P75	72.00–86.00	68.99–81.00	
Sleepiness			
Median	9.00	13.00	**0.001**
IQR	6	8	
P25-P75	7.00–13.00	8.00–16.00	
Empathy			
Median	118.00	121.00	0.185
IQR	16	12	
P25-P75	110.00–126.00	114.00–126.00	

Mann - Whitney test. *P* values marked in bold indicate a statistically significant difference (*p* < 0.05)

Female residents had significantly lower scores for quality of life in the domains of time management (30.3, females vs 41.1, males; *p* < 0.001), psychology (48.1, females vs 56.7, males; *p* < 0.01) and physical health (42.8, females vs 53.6, males; *p* < 0.05) than those for male residents (Table 2).

**Table 2:** VERAS-Q quality of life domains scores of medical residents according to gender

Domains of VERAS-Q	Gender		*P*
	Male (*n* = 78)	Female (*n* = 31)	
Time management			
Median	41.07	30.35	**0.000**
IQR	16.51	17.85	
P25-P75	32.26–51.78	25.00–42.85	
Psychological			
Median	56.73	48.07	**0.001**
IQR	19.71	15.38	
P25-P75	46.15–65.86	38.46–53.84	
Learning environment			
Median	57.35	54.41	0.067
IQR	16.54	8.82	
P25-P75	51.47–68.01	50.00–58.82	
Physical health			
Median	53.57	42.85	**0.013**
IQR	25.89	25.00	
P25-P75	42.85–68.75	35.71–60.71	

Mann - Whitney test. *P* values marked in bold indicate a statistically significant difference (*p* < 0.05)

Significant gender differences were observed regarding positive perceptions of quality of life on the VERAS-Q (Table 3). Male residents had more positive perceptions than female residents in certain domains, such as those related to time management “I can manage my time well” and “My medical residency activities are hard for me”, perception of the learning environment “My quality of life at medical residency is good” and “I feel valued for my work in the residency program”, and psychology “I feel safe to carry out my activities in the residency program”, “I can’t absorb the content”, “I haven’t been able to properly concentrate lately”, “I get stressed in my residency program” and “My vitality is enough to do my activities in the medical residency”.

**Table 3:** Frequency of answers to VERAS-Q questions according to gender

	% with a positive quality of life perception		Difference between male and female residents	
	Male	Female	Ratio	Absolute difference
50. I feel safe to carry out my activities in the residency program.^#^	76.90	38.70	1.99	38.20
23. I can manage my time well.^#^	46.20	12.90	3.58	33.30
39. I can’t absorb the content.^#^	70.50	41.90	1.68	28.60
42. I haven’t been able to properly concentrate lately. ^#^	43.60	16.10	2.71	27.50
25. My quality of life at the medical residency is good.^*^	48.70	22.60	2.15	26.10
43. I get stressed in my residency program.^#^	32.10	6.50	4.94	25.60
38. My vitality is enough to do my activities in the medical residency.^*^	73.10	48.40	1.51	24.70
45. I feel under pressure by having to financially depend on my family.^*^	46.20	22.60	2.04	23.60
48. I fell valued for my work in residency program.^*^	33.30	9.70	3.43	23.60
18. My medical residency activities are hard for me.^*^	59.00	35.50	1.66	23.50
8. I have time for extracurricular activities.	47.40	25.80	1.84	21.60
32. I can’t take care of my looks.	43.60	22.60	1.93	21.00
37. My own expectation worsens my quality of life.	37.20	19.40	1.92	17.80
20. I have good access to medical care.	42.30	25.80	1.64	16.50
6. My residency environment is health.	51.30	35.50	1.45	15.80
19. I have enough time to study.	21.80	6.50	3.35	15.30
4. I have time for my family.	28.20	12.90	2.19	15.30
13. The contact with my patients increase my quality of life.	69.20	54.80	1.26	14.40
51. My work load on the residency program decreases my quality of life.	20.50	6.50	3.15	14.00
1. My quality of life is good.	60.30	48.40	1.25	11.90
40. I can properly eat.	37.20	25.80	1.44	11.40
27. I have been feeling down lately.	50.00	38.70	1.29	11.30
34. I have been feeling anxious lately.	33.30	22.60	1.47	10.70
36. I am happy about my love life.	71.80	61.30	1.17	10.50
41. I regularly do physical activities.	42.30	32.30	1.31	10.00
17. I have time for cultural activities.	32.10	22.50	1.43	9.60
44. I am satisfied with my housing condition.	83.30	74.20	1.12	9.10
30. I am satisfied with my residency program.	66.70	58.10	1.15	8.60
3. I get supervision in my practice.	50.00	41.90	1.19	8.10
21. Most of my residency classes are bad.	46.20	38.70	1.19	7.50
29. I have enough sleeping time.	19.20	12.90	1.49	6.30
31. I have time for my friends.	28.20	22.60	1.25	5.60
2. I don’t make the most of my life.	33.30	29.00	1.15	4.30
35. My residency environment is competitive.	26.90	22.60	1.19	4.30
7. I have a good relationship with my residency colleagues.	97.40	93.50	1.04	3.90
11. My life makes sense.	91.00	87.10	1.04	3.90
26. My medical residency deprives me of some personal appointments.	3.80	0.00	–	3.80
16. Don’t take care of my health.	35.90	32.30	1.11	3.60
5. Sometimes I feel humiliated in the medical residency.	67.90	64.50	1.05	3.40
49. Work in an interdisciplinary team worsens my quality of life.	83.30	80.60	1.03	2.70
14. I push myself too much in my medical residency.	21.80	19.40	1.12	2.40
28. I have a good access to psychological care.	11.50	9.70	1.19	1.80
15. I am pushed a lot by my preceptors/supervisors.	62.80	61.30	1.02	1.50
33. My family expectation towards my performance decreases my quality of life.	62.80	61.30	1.02	1.50
9. I don’t have enough free time.	20.50	19.40	1.06	1.10
12. My relationship with my preceptors/supervisors is good.	88.50	90.30	0.98	−1.80
22. My relationship with my other year residency colleagues is good.	88.50	93.50	0.95	−5.00
46. To have another professional activity besides medical residency improves my quality of life.	20.50	25.80	0.79	−5.30
10. My faith improves my quality of life.	46.20	51.60	0.90	−5.40
47. Hierarchy between residents of my residency program worsens my quality of life.	78.20	90.30	0.87	−12.10
24. My health is good.	61.50	74.20	0.83	−12.70

Fisher’s test. ^*^P < 0.05 ^#^*P* < 0.01

Regarding other statements, female residents had more positive perceptions than male residents, although these differences were not significant. For example, in the domain of perception of the learning environment, female residents perceived the following statements more positively: “My relationship with my preceptors/supervisors is good”, “My relationship with my other year residency colleagues is good” and “Hierarchy between residents of my residency program worsens my quality of life”. Similarly, female residents had more positive perceptions in the psychological domain to “To have another professional activity besides medical residency improves my quality of life” and “My faith improves my quality of life” and in the physical domain to “My health is good”.

Although the scores concerning resilience did not differ between genders, some significant differences were observed regarding the responses to the questionnaire (Table 4). In the domain related to meaning in life, significant gender differences were observed for the statement “I feel proud to have accomplished things in my life,” with 94% agreement among male residents, while 80.6% of female residents expressed agreement with this perception (*p* < 0.05). In the serenity domain, significant differences were found for the statement “When I make plans, I follow through with them” (*p* < 0.05). In the self-sufficiency domain, significant differences were observed for the affirmation “I am friends with myself”, with 88.5% agreement among male residents and 71.0% agreement among female residents (*p* < 0.05).

**Table 4:** Frequency of answers to RS-14 questions according to gender. by sex. Fisher’s test

	Gender			
	Male		Female	
	Disagree + Indifferent	Agree	Disagree + Indifferent	Agree
	N (%)	N (%)	N (%)	N (%)
I usually manage one way or another.	8 (10.3%)	70 (89.7%)	3 (9.7%)	28 (90.3%)
I feel proud that I have accomplished things in my life.^*^	4 (5.1%)	74 (94.9%)	6 (19.5%)	25 (80.6%)
When I make plans I follow through with them.^*^	14 (17.9%)	64 (82.1%)	12 (38.7%)	19 (61.3%)
I am friends with myself.^*^	9 (11.5%)	69 (88.5%)	9 (29.0%)	22 (71.0%)
I feel that I can handle many things at a time.	29 (37.2%)	49 (62.8%)	12 (38.7%)	19 (61.3%)
I am determined.	10 (12.8%)	68 (87.2%)	2 (6.5%)	29 (93.5%)
I ca get through difficult times because I’ve experienced difficult before.	18 (23.1%)	60 (76.9%)	6 (19.4%)	25 (80.6%)
I have self-discipline.	19 (24.4%)	59 (75.6%)	13 (41.9%)	18 (58.1%)
I keep interested in things.	13 (16.7%)	65 (83.3%)	10 (32.3%)	21 (67.7%)
I can usually find something to laugh about.	14 (17.9%)	64 (82.1%)	5 (26.3%)	26 (83.9%)
My belief in myself gets me through hard times.	23 (29.5%)	55 (74.3%)	12 (38.7%)	19 (61.3%)
In an emergency I’m someone people generally can rely on.	13 (16.7%)	65 (83.3%)	6 (19.4%)	25 (80.6%)
My life has meaning.	10 (12.8%)	68 (87.2%)	3 (9.7%)	28 (90.3%)
When I’m in a difficult situation, I can usually find my way out of it.	4 (5.1%)	74 (94.9%)	1 (3.2%)	30 (96.8%)

Fisher’s test. ^*^P < 0.05

The results of the qualitative evaluation were divided into five sections. In the following sections, each theme is discussed with pertinent quotations.

1. Perceptions of medical resident quality of life

Female medical residents perceived quality of life as a multidimensional construct comprising life satisfaction, freedom, time management, healthy habits, social and affective relations and the balance between work and leisure time (Table 5). Participants reported the following:

*“Quality of life in my view is when you are able to do what you like to do, what you want to do. When your dreams come true.”*

*“Have time to be something other than being resident.”*

(2) Factors that increase resident quality of life

**Table 5:** Categories and issues for the theme of “Perceptions of quality of life among female medical residents”

Category	Issues	Examples
Satisfaction/happiness	Achieving aims Positive view of life	“I think it comes from being happy, happy and fulfilled in what you’re doing. Like at work, and at residency. I think coming home feeling good, feeling that you were supported, feeling that you achieved what you wanted to achieve.” “It’s you searching for a little bit of happiness in your day, like, you get home and feel good and feel happy. For me, I think this is the concept of quality of life.”
Balance/harmony	Work and leisure in harmony	“Quality of life is being able to fulfill your obligations, like, as a citizen who can determine their financial life, but at the same time you’re able to enjoy your leisure, without one thing affecting the other, and while maintaining a balance between the two.”
Health	Sleep Eating habits	“Quality of life is getting a chance to sleep.” “Quality of life is getting through the residency without having to suffer discomfort because of it.”
Free time	Leisure, relationships, study, physical activity	“To have quality of life you need to have time to do what you like.”
Interrelation of several life aspects	Social, affective, work and health	“Quality of life is doing things that I enjoy, having time for myself, having time to do things I like, for my family and also having quality of life at work.”

Female residents associated good relationships with a multidisciplinary team, adequate supervision, free periods during the internship and the feeling of usefulness in patient care as academic factors associated with improved quality of life.

Female residents also believed that they are more empowered and have greater autonomy when managing their time. They reported new and strong relationships between members of the work group as highly important to improved quality of life (Table 6).

*“For me, it was a relief to leave home and have my own time, my silence and do things at my pace, even if, um, it forces me to go to the shops and take care of things, like, for me, this so improved my quality of life.”*

(3) Factors that decrease medical resident quality of life and coping strategies

**Table 6:** Categories and issues for the theme of “Factors that increase resident quality of life”

Category	Issues	Examples
Feeling useful	Patient recognition	“But at the same time, my sense of gratification depends a lot on the patient, being able to do what little I can to improve their quality of life, for me, already helps a lot.”
Significant relationship	Friends, boyfriend/girlfriend, family	“But when we come here, the relationships we create here also help, and our group is pretty, pretty cool peeps.”
Adequate Internship	Adequate supervision/distribution of time Free period in the palliative care internship	“It’s knowing that there is someone for you to go and discuss things with, talking helps, it’s a relief, it’s gives you much needed tranquility.” “So you don’t get overloaded, don’t have to attend too many patients in the outpatient clinic. You have time for lunch.” “Look, I dream about my weekly free period, ah my free period, my free period.”
Work environment	Adequate relationship with multidisciplinary team	“With the multi-team, I do everything to get along. Because when I get along I'm very happy.”
Empowerment	Own space	“For me, it was a relief to leave home and have my own time, my silence and do things at my pace, even if, um, it forces me to go to the shops and take care of things, like, for me, this so improved my quality of life.”

Factors that most diminished female residents’ quality of life were associated with the learning environment; work overload; poor supervisors, coordinators, and preceptors; infrastructure; distant relationships during residency with colleagues and family; and sleeping problems due to night shifts and lack of structure in on-call rooms.

Female residents also reported difficulty coping with feelings of attachment to patients, insecurity, guilt, time management, some aspects of professional ambition and personal demands. (Table 7)

*“We give up a lot of things. I think in our profession, through our choices, we end up having to give up a lot of things that I think are important.”*

*“I think there is a lot of expectation on us, so we have to be very, like, up to date, and it turns out we don’t have a lot of time for other people, and that’s very annoying, you know.”*

(4) Differential factors that may affect quality of life during medical residency for men versus women

**Table 7:** Categories and issues for the theme, “Factors that decrease medical resident quality of life”

Category	Issues	Examples
Human resources	Teachers Managers	“The problem is more that it seems they don’t add anything. Most of the time you’d be better off solving the cases on your own than looking for supervision. But it ends up affecting you anyway, because at least you leave there feeling that “ah, who knows, I could have done something better, or done something different.” “We don’t feel the partnership between this preceptor group and the residents.”
Moral abuse	Medical assistants/ Multidisciplinary team	“Sometimes we’re treated badly by the nursing staff, and the team.” “Likewise assistants, there are some good assistants, but jeez, some assistants just humiliate you, even though they don’t know what you’ve been through that day.”
Demands	Personal, institutional	“I demand a certain level of perseverance from myself, I demand too much of myself sometimes.” “These here are the best students from the best colleges in Brazil – this already creates a certain expectation that, maybe if you didn’t have any, you got it right away. And it’s very annoying, you’re always being treated like the best of the best, we see this a lot in our residency.”
Financial resources	Financial dependence	“This is stressing me out right now, because before I had money that I saved the whole year that I was in the Navy. And then last year, I chose not to work and I’ve spent all the money I had. And now it's running out, and I'm having to go back to shiftwork, and so I’m trying to get back into the schedules.”
Time	Difficulty in time management	“We still don’t know how to manage our own time, but we’ll learn to do it during our lives. Nowadays we still don’t know how to say “no,” we’re beginning to figure it out, but we still don’t know how to say “no.” I guess we’ll learn over time.”
Reduced freedom	Relationship/leisure Required activities	“We give up things, I've given up certain relationships, I think people, I think especially for women, it’s very hard dating someone who isn’t a doctor.” “And so, I’ve felt a little of this, not being able to pay attention to my relatives, because I had to attend to other people, other sick people. So that, I suffer from this a little.”
Distant relationships	Residency colleagues/ Family/ Loneliness	“In the clinical residency at the hospital, people are very much in it for themselves only.” “So, you come home at night and have no one to share with, I miss that a bit.”
Sleep	Night shifts Lack of structure	“What annoys me is night shifts, it’s not sleeping at home.” “For me, the room is bad, it's completely filthy, and I’m really allergic.”
Lack of attention to health	Eating	"Having a short lunch hour annoys me as well, worse yet, if I can’t have lunch. It’s disrespectful not to have lunch break, it’s just not on.”
Work overload	Number of patients Workload Multiple tasks Multidisciplinary team inefficiency	“I also think volume, work overload really lowers the quality of life. Because I feel like I’m not being the best doctor for that type of patient.” “All the time I need to keep returning to places to check whether what I’d requested had been done.”
Changes of environment	Changes of city Loss of previous infrastructure Changes in responsibility	“Here people are colder. So, I suffered. I suffered. I called my mom. And I cried.” “So the change for me from student to resident what was most, the most difficult part was the responsibility. For me, the hardest, the most difficult sometimes was dealing with powerlessness or people’s deaths, however much we do everything we possibly can, a lot of people die.”
Professional actuation	Acting without technical knowledge	“And so, that consumed me a lot; I had to talk about palliative care, without ever having seen palliative care.”
Difficulty dealing with feelings	Attachment to the patient Insecurity Guilt	“I get very attached, I have this problem. I get very attached not only to the family, but I get very attached to the patients. Then I suffer so much with each loss.” “Help me? I'm suffering a bit this year with that. Because I’m not sure what I want to do.” “I went to a wedding and it was like a week later. And we commented – jeez, but it was so good, but I felt so guilty, stopping for the weekend on the eve of the test, being able to go and do something other than the residency.”

Among the factors that may affect gender differences on quality of life, female residents cited internal/societal and cultural demands; biological factors; women having a higher perception of their environment, being more expressive of their feelings, being more attached to patients than male residents; and having domestic responsibilities, such as caring for family and personal life planning (Table 8).

*“Being in the residency was really cool because I had the chance to better understand the male universe, and it’s simpler, they are simpler. Sometimes I think women perceive many things at the same time, and that sometimes men don’t have that, and perhaps this is why women suffer more with many things.”*

(5) Proposals to improve female resident quality of life

**Table 8:** Categories and issues for the theme, “Differential factors that may affect quality of life during medical residency for men versus women”

Category	Issues	Examples
Demands	External Institutional Societal Internal Personal life planning	“For a woman, it’s like constant, more is demanded of her than men, because, er, she has to be pretty, she has to smell good, she has to be well-dressed, she has to be nice. So, I think this exists and for a man, I think it’s more flexible.” “I feel that the residency itself doesn’t create a difference, it doesn’t distinguish between men and women, I think it's something that comes more from us.” “I think women care more, so they’re more concerned about the relationship in the sense of building a family. The guys only focus on their career, but women have their careers, their family “Oh, tidying up the house... I doubt if a man would waste time cleaning the floor and cleaning the bathroom.”
Biological factors	Menstrual period	“Oh, I also think the hormones are terrible!" “I think that, with PMS, quality of life gets worse ...”
Perception	Critical perception	“I think that women sometimes suffer more, are more into details, can perceive things better.”
Feelings	Expression Scrutinize Attachment to patients	“Men are less complicated, for sure, they don’t think about numerous things, men externalize less.” “I think women are much, much more sensitive, I think they suffer more, I think they get more attached. The threshold is much lower, like, I don’t see men... few of the male residents in our group care, as I see it, so, they don’t care, er, don’t get shaken.”
Relationships	Formation of groups	“They show greater facility at socializing, at dealing with differences, with everything. It’s something intrinsic to men, because with us, they really don’t even listen to what we say.”

To improve quality of life, female residents proposed having better supervisors and teaching methodologies, mentorship programs for those interested, improved infrastructure regarding on-call rooms, time for meals, improved computer systems and increased numbers of professionals on multidisciplinary teams (Table 9).

*“An adequate on-call room, wow, that makes a big difference.”*

**Table 9:** Categories and issues for the theme, “Proposals to improve quality of life for future internal medicine residents”

Category	Issues	Examples
Infrastructure	On-call room Bathrooms Socialization space Eating	“An adequate on-call room, wow, that makes a big difference.” “A clean bathroom would be amazing. Having a bathroom, wow. Seriously. Very nice.” “There could be an area that was half for socializing and half library.” “Improve the food! “Better food over at the Palhetas restaurant.”
Tutoring	Adequacy for internship	“Maybe R2 are the ones who most need the tutoring, but they have the least time to go.”
Teaching methodology	Non-compulsory theory classes	"An important class that explained the new guidelines. These are things that we have to keep up-to-date on.”
Human resources	Multidisciplinary team Supervision	“Hire more nurses.” “I’d invest in training on nursing techniques.” “The dull stint in the ER, if you had someone good to discuss the cases, who brought me relevant information with current scientific knowledge, awesome.”
Hospital functioning	Computer system Decreased bureaucracy	“The system. Fix the computers. Man, the system drags us down. Seriously, not good enough. The system wastes a lot of time. The thing is, we could be with the patient, but we waste a lot of time in the system, you know. It’s a ton of bureaucracy to solve. So, if I could spend money...”

## Discussion

In this study, we analyzed gender differences in the perception of quality of life, with quantitative methods, during internal medicine training via a group of first-year internal medicine residents in a Brazilian medical school. We also attempted to understand the difficulties encountered by female medical residents during this critical period of medical education using a focus group analysis.

The demographics of our study showed that females comprised 28.4% of the analyzed trainees, highlighting the persistent male predominance in the internal medicine specialty at our faculty of medicine in Brazil.

We observed significant gender differences regarding quality of life specific to medical residency, as assessed by the VERAS-Q. Female residents exhibited significantly lower scores for quality of life in the domains of time management, psychology and physical health than male residents. Previous studies on medical education also demonstrated high levels of academic stress and worse well-being among female medical students [2, 3, 39].

Many factors, such as the student-residency transition period, professional responsibility, social isolation, fatigue and sleep deprivation, are associated with psychological reactions and negative behaviors, such as a depressive state, making resident physicians a vulnerable group for emotional disorders [40]. In the qualitative evaluation, we found that female medical residents perceived more suffering from greater emotional attachment to the patients and greater difficulty in forming new affective bonds than male residents. Regarding fatigue and sleep deprivation, our results showed that female residents had significantly higher scores for daytime sleepiness than male residents, with values considered at the pathological somnolence level. A previous study found high daytime sleepiness scores in a group of medical residents, with the highest values among female residents [41]. Our data demonstrate why the factors associated with these psychological and behavioral reactions are more impactful in the female group.

We found that female residents had lower quality of life scores in the time management domain, which were elaborated on in the focus group analysis where they reported feeling overwhelmed with medical tasks and stressed in their residency program. Similar findings were shown in a study on the well-being of residents in Alberta, Canada, which found that 40% of female residents reported significant levels of stress, whereas only 27% of male residents had similar levels of stress [3]. Moreover, that study disclosed a difference in the sources of stress, with 79% of female residents reporting that their workload was a source of stress compared with 64% of male residents [3].

According to the qualitative data in our study, female residents expressed feelings of guilt and stress because they did not have time to dedicate themselves to family, friends and other leisure activities. The difficulty with time management is that this practice is stressful; feelings of lost time dedicated to family, friends and leisure cause emotional exhaustion, and these combined factors diminish quality of life among female residents. Among resident physicians, sacrificing time with family members, leisure friends or any other activities for medical residency responsibilities may lead to burnout [42]. Ríos et al. [43] assessed stress levels among medical residents and observed that stress interferes with family relationships.

In the focus group analysis, the residents’ reports revealed the concern among women of having to dedicate themselves to personal life planning and internal and external demands, which reflect the continuation of traditional historical models that overwhelm women. This conflict between work and personal life has been reported in the literature, considering that most women contemplate the possibility of marriage and children at some point in their careers [44].

Rojas’ [45] concept of quality of life is an eminently human characterization and is linked to the degree of satisfaction found in the personal, family, love, social and environmental life of the individual. Faced with task overload, maintaining the balance between one’s personal and professional life is more difficult for women to manage, which generates a feeling of dissatisfaction and the perception of a worse quality of life [46].

Our study also showed that women have a more critical perception of the educational environment, showing more disapproval of the situations encountered and being more expressive of the problems. These intrinsic female factors may have influenced the findings from the questionnaires. The gender differences might be attributed to the greater sensitivity of women, who usually have a more refined perception of quality of life and a greater chance of identifying negative concerns compared to males [2, 47].

Autonomy, goal achievement, confidence and a sense of increasing mastery are associated with greater resident well-being [18]. In our focus group analyses, female residents disclosed insecurity and difficulties in concentration and knowledge acquisition. Previous studies reported that women tended to underestimate their abilities [48–50], and this lack of confidence may induce stress [51] and psychological vulnerability [48], which can impact perceived quality of life.

Because quality of life depends on personal and environmental factors, we evaluated resilience as an intrinsic trait of the individual that could influence quality of life. Some studies have shown that resilience levels were associated with a higher perceived quality of life [10, 52, 53]. Resilient students have a better perception of quality of life, both in positive and negative educational settings [10]. Surprisingly, our study did not show gender differences regarding resilience levels. However, gender differences were found when analyzing the responses to the resilience questionnaire by item, with worse scores for women.

Another intrinsic trait examined in our study was empathy. Despite gender differences in the response patterns to the quality of life assessment, we did not find gender differences in empathic abilities. Shanafelt et al. [12] observed that resident mental well-being correlated with empathy. Paro et al. [54], in a study on the relationship between empathy and quality of life among medical students, observed that female students showed greater empathic consideration than male students in the emotional dimensions of empathy and that the differences were maintained over the duration of the undergraduate medical course. However, the authors did not identify gender differences for the cognitive dimension of empathy, which involves the willingness to adopt the perspective of another [54]. The difference between these authors’ findings and our results might be related to differences in the instrument used to assess empathy and because the self-assessment questionnaire might have induced socially acceptable responses [55]. Another explanation for this difference is that the female residents’ empathy may have decreased during the first year of residency. Haar, Halitsky and Stricker, [56] suggested that women could become less compassionate during training for this male-dominated profession to prove themselves worthy. In contrast, other studies suggested that female physicians have higher empathy ratings than men [57, 58] due to their increased receptiveness to emotional signals [59]. Another possibility is that male residents from this study might have achieved higher empathy ratings resulting from the interpersonal skills training emphasis of this internal medicine residency program [60].

Studies on coping have shown that residents have great difficulty coping with poor working conditions [61]. In our study, female residents suggested proposals to improve infrastructure conditions as being important for improving their quality of life.

Greater social support from family, friends or physician support groups was associated with decreased emotional exhaustion, depersonalization and increased personal accomplishment [18]. The female residents in the focus group analysis suggested that the development of activities that might enhance relationships between residents and might help them expand their social support circles. The implementation of other supportive programs for residents may improve both personal and professional quality of life [62]. Our residents indicated that they should learn to seek help when they feel the need and raised the issue of mentoring as important for psychological support. They also suggested that mentoring be included in the residency program, with elective participation, to discuss challenges encountered during the training period.

The findings from this study should be considered in light of its advantages and limitations. The strategy of administering the questionnaires immediately before the OSCE and thus associated with the “paper and pen” methodology favored the adherence and participation of the residents, yielding a response rate of 90.83%. Alternatively, the design of the study may have affected the results because the questionnaires were applied immediately before the academic performance evaluation via the OSCE model, which is a stress factor for the participants [63]. Furthermore, the sample analyzed is considered one of convenience because it was from a single medical residency program and a single specialty.

## Conclusion

Female residents had lower quality of life scores in the time management, physical health and psychological domains than male residents. The percentage of pathological scores in the daytime sleepiness assessment were higher for female residents. Women residents disclosed time management difficulty in striking a balance reported concentration and knowledge acquisition difficulty, insecurity, feelings of loss, more critical perception and a struggle to create affective bonds. Measures suggested by the female medical residents to improve quality of life during this medical training period include investing in mentoring, improving time management and encouraging activities that favor the development of relationships.

## Abbreviations

CASP: Critical Appraisal Skills Program
IQR: Interquartile range
JSE: Jefferson Scale of Physician Empathy
OSCE: Objective Structured Clinical Examination
RS-14: Wagnild & Young Brief Resilience Scale
VERAS-Q: Questionnaire to evaluate quality of life specifically in medical residents
